# A lightweight hybrid deep learning framework for multi-pill detection, multi-attribute recognition, OCR-based imprint analysis, and metadata retrieval

**DOI:** 10.3389/frai.2026.1890296

**Published:** 2026-07-20

**Authors:** A. Sai Sachin, J. Karthikeyan

**Affiliations:** School of Computer Science Engineering and Information Systems, Vellore Institute of Technology, Vellore, Tamil Nadu, India

**Keywords:** clinical decision support, computer vision, deep learning, medication safety, multi-pill detection, optical character recognition, pharmacovigilance, pill recognition

## Abstract

**Introduction:**

Adverse drug events (ADEs) remain a major cause of preventable healthcare complications due to incorrect pill identification, dosage errors, and confusion between visually identical pills, particularly among older adults, visually impaired individuals, and people with limited health literacy. Recent advances in artificial intelligence and computer vision have enabled automated pill recognition systems. However, many existing methods address detection, classification, and imprint recognition as isolated tasks without providing a lightweight unified framework suitable for real-time healthcare deployment.

**Methods:**

This study presents a lightweight hybrid framework for multi-pill and multi-attribute recognition to support pharmacovigilance and artificial intelligence-driven clinical decision support. The proposed framework integrates pill detection using YOLOv26n, MobileNetV4-ConvSmall-based classification, EasyOCR-based imprint recognition, and metadata retrieval into a unified prediction pipeline. A customized benchmark dataset containing 400 pill classes, each with 50 images, for a total of 20,000 images, was used for classification. Each image has two pills, comprising 40,000 manually labeled pill instances used for detection. Comparative experiments, functional comparisons with existing pill recognition systems, and cross-device qualitative evaluations were conducted to assess framework performance, robustness, and deployment feasibility.

**Results:**

YOLOv26n achieved a precision of 0.963, a recall of 0.982, mAP@50 of 0.989, and mAP@50-95 of 0.981, while MobileNetV4-ConvSmall achieved a class accuracy of 99.60% with higher performance in pill shape and color accuracy. Comparative optical character recognition (OCR) analysis shows that EasyOCR achieved more reliable imprint recognition performance than TesseractOCR and TrOCR. Comparative framework evaluation demonstrated that the YOLOv26n with MobileNetV4-ConvSmall combination achieved the high overall recognition performance, obtaining a Top-1 accuracy of 89.83% and a Top-5 accuracy of 99.49%. Cross-device qualitative evaluation using smartphone cameras and direct laptop uploads demonstrated robust performance under varying image noise conditions.

**Discussion:**

The proposed framework demonstrates the potential of lightweight deep learning architectures for scalable medication identification and AI-assisted pharmacovigilance applications. By integrating detection, classification, imprint recognition, and metadata retrieval into a unified framework, the proposed system has the potential to support medication identification and improve assistive healthcare workflows. The final clinical judgment and medication verification should remain under the supervision of a healthcare professional.

## 1 Introduction

Adverse drug events (ADEs) remain a significant source of preventable healthcare complications and continue to contribute crucially to medication-based morbidity worldwide (Mekonnen et al., 2018). Medication errors often arise from incorrect pill identification, confusion between visually identical pills, improper dosage management, and inadequate communication between patients and healthcare professionals (Unnissa et al., 2024). Such errors may result in adverse drug reactions (ADRs) (Montané and Santesmases, 2020), harmful drug-drug interactions (Feng et al., 2020), organ toxicity, and reduced treatment effectiveness. Vulnerable populations, including older adults, visually impaired individuals, and people with limited health knowledge, are susceptible to medication-based risks (Lavan and Gallagher, 2016). Consequently, improving medication safety and minimizing identification errors have become important priorities in modern healthcare systems.

Advancements in artificial intelligence (AI) (Wang et al., 2026a), deep learning (DL) (Halomoan et al., 2026), and computer vision (CV) (Khadem et al., 2026) have enabled automated visual recognition systems that achieve high accuracy across a range of medical imaging and healthcare applications (Albuquerque et al., 2025). The deep learning methods have been widely investigated for pill detection, classification, and imprint recognition in pharmaceutical informatics. Lightweight convolutional neural networks (CNNs) (Nguyen et al., 2025) enable effective classification of visually similar pills, and object detection architectures enable the localization of pills in complex scenarios. In addition, based on the imprint evaluation, the optical character recognition (OCR) (Suntronsuk and Ratanotayanon, 2017) approach provides textual information, improves pill imprint recognition, and minimizes confusion during pill identification.

Although these advances have been made, many existing pill recognition systems use detection, classification, imprint recognition, and metadata retrieval as independent procedures rather than as components of a integrated end-to-end framework. Several studies focus on isolated technical performance, with a limited combination of pharmacovigilance (Bernaus and Bournissen, 2026) and AI-driven clinical decision support systems (Elhaddad and Hamam, 2024). In addition, real-world deployment complexity remains insufficiently analyzed, including multi-pill recognition, visually identical pill differentiation, cross-device evaluation, lightweight inference, and reliable metadata retrieval. The lack of computational efficiency frameworks that simultaneously address these challenges remains a gap in the current study.

Medication detection of pills in real-world conditions presents various visualization and computational challenges. Pills often have highly identical appearances in shape, color, texture, and imprints, which can cause confusion during self-medication or the prescription process (Hamid et al., 2022). It is important to identify medications based on multiple visual features, such as the class, shape, and color of the pills. In addition, a reliable and lightweight model is necessary for real-world deployment under variable factors such as illumination, camera, view angle, and input device (Ghassab-Abdollahi et al., 2024). Robust cross-device evaluation is necessary for practical implementation in edge-based devices and mobile healthcare systems.

To address these challenges, this study proposes a lightweight hybrid deep learning framework for multi-pill detection and multi-attribute classification designed to support medication safety, pharmacovigilance, and AI-assisted clinical decision support systems. The proposed framework integrates YOLOv26n-based pill detection (Chakrabarty, 2026), MobileNetV4-ConvSmall-based multi-attribute classification (Qin et al., 2024), EasyOCR-based imprint recognition (Salehudin et al., 2023), and metadata retrieval (Li and Kleinert, 2023) within a unified prediction framework. The system performs simultaneous pill localization, classification, imprint analysis, and contextual metadata retrieval to improve robustness under real-world conditions. In addition, computationally efficient architectures were selected to improve scalability and support possible edge-device deployment.

A customized benchmark dataset comprising 400 pill classes, each class with 50 images, and 20,000 images was utilized for classification experiments. Each image contains two pills, yielding approximately 40,000 manually annotated pill instances for object detection training. Multiple detection and classification architectures were comparatively evaluated to identify an optimal balance between computational efficiency and predictive performance. Additional experiments were conducted, including quantitative framework comparisons, a capability-based analysis of existing pill recognition systems, and cross-device qualitative evaluation, to assess the robustness and practical applicability of the proposed framework. Cross-device experiments using smartphone cameras and direct laptop uploads further evaluated the system's stability under heterogeneous imaging conditions.

The major contributions of this study are summarized as follows:

- A lightweight end-to-end multi-pill recognition framework is developed to integrate multi-pill detection (Nguyen et al., 2023), multi-attribute classification (Danas et al., 2006), imprint recognition, and metadata retrieval into a single recognition pipeline.
- A manually labeled customized benchmark dataset containing approximately 40,000 pill images in 400 pill variants to support large-scale multi-pill recognition healthcare system.
- Integration and comparative evaluation of multiple OCR models, including EasyOCR, TesseractOCR, and TrOCR, for reliable recognition of the pill imprint under various imaging conditions.
- Additional cross-device (Hoffman et al., 2022) qualitative evaluations were conducted in multiple image acquisition conditions to assess framework reliability.
- A comparative performance analysis of multiple lightweight framework configurations was performed to evaluate the influence of different detection and classification architectures on overall pill recognition performance, this supports practical edge-based deployment in healthcare environments (Hartmann et al., 2022).

## 2 Related work

### 2.1 Pill detection approaches

Several studies have examined object detection using deep learning methods for the automatic detection of pills. Current YOLO-driven models have shown robust real-time object detection capabilities with stable localization. The YOLOv8-based pill detection model achieved precision and mean average precision values of 0.99, indicating reliable real-time detection capabilities (Qomariah et al., 2026). Improvements to the YOLOv8 model, incorporating SPD-Conv, the BiFormer attention mechanism, and the circular smooth label, achieved a precision of 94.24% and a mean average precision of 94.16% for detecting small rotating objects in pharmacy settings (Pan et al., 2025). The lightweight deployment-driven pipeline integrating YOLOv8 with ShuffleNetV2 and pruning strategies achieved a mAP of 95.1% while reducing computational complexity for embedded systems (Sun et al., 2024).

However, these methods achieve strong localization performances; most detection models focus on pill counting or localization and do not integrate other important modules, such as pill classification, imprint recognition, and metadata retrieval. Additionally, many pipelines were evaluated under less experimental conditions, with insufficient reliability for multi-pill conditions.

Multimodal frameworks that integrate Fast R-CNN, YOLOv5, and graph-based methods have been proposed to demonstrate the complexity posed by visually similar pills (Nguyen et al., 2023). However, the improved recognition capabilities of these models pose considerable architectural challenges and do not incorporate an OCR-driven imprint recognition phase. A two-stage Mask R-CNN-driven framework examined multi-pill detection using automatic labeling and augmentation (Kwon et al., 2021). However, these remain computationally intensive and offer limited generalization in challenging real-world imaging scenarios.

### 2.2 Pill classification approaches

The classification of pills in healthcare applications increasingly relies on convolutional neural networks and methods for visual feature extraction. Attention-based models incorporating contour, texture, RGB, and textural features achieved 96.23% of accuracy and were deployable in edge devices (Nguyen et al., 2026). An automated pill classification framework, built on a MobileNet-based medication identification system, achieved a 98% accuracy by combining visual attribute classification with database-matching techniques (Jha et al., 2025).

Despite promising performance in classification, current methods primarily focus on isolated visual classification and lack integration in multi-pill localization, OCR-driven imprint recognition, or metadata retrieval. In addition, many current methods rely heavily on shape and color features, which limit their robustness when differentiating visually identical pills.

### 2.3 OCR-based imprint recognition

Optical Character Recognition (OCR) approaches have proven crucial to pharmaceutical informatics for recognizing imprint information on healthcare packages. In a dual-engine system using both CnOCR and EasyOCR, including preprocessing and field extraction, the character-level accuracy was 97.1% (Wu and Chang, 2025), while the F1-score was 94.0%. A new geometry-based system that utilizes textSnake extraction, geometric alignment, and ABINet-based OCR improved detection accuracy for curved and embossed imprint patterns (Jo et al., 2026).

Although these methods highlight strong textual recognition capabilities, most OCR-based systems focus primarily on text extraction rather than unified pill recognition. OCR performance may also degrade under challenging imaging conditions, such as low illumination, reflective surfaces, occlusions, and degraded imprint visibility. In addition, OCR-driven healthcare applications using EasyOCR and similarity matching highlighted strong pharmaceutical label retrieval performance, but remained largely text-oriented without integration of visual detection and classification modules (Ghazaryan et al., 2025).

### 2.4 AI-driven medication identification systems

Recent studies have attempted to integrate pill recognition into broader AI-based healthcare and biomedical informatics frameworks. Hybrid frameworks integrating YOLOv12n-based pill detection with ConvNeXt-Tiny-driven multi-head classification achieved a detection precision of 0.972 with strong classification performance for a medication safety application (Sachin and Karthikeyan, 2026). However, imprint recognition and cross-device qualitative evaluation were not incorporated into the framework.

Additional multimodal systems integrated YOLOv7 with latent diffusion enhancement for multi-pill recognition, achieving approximately 97% single-pill recognition accuracy while improving recognition stability for overlapping medications and visually challenging scenes (Lee et al., 2025). Nevertheless, these systems failed to integrate OCR-based imprint recognition and metadata-supported recognition workflows.

Various imaging strategies have been examined to differentiate medications with similar features. The application of infrared imaging combined with enhanced YOLOv5s networks proved to be more effective at recognizing pills in complex imaging conditions, achieving a mAP@50 of 89.30% (Wang et al., 2024). Such solutions require specialized imaging equipment, while the datasets used for testing were relatively small, thereby limiting the scalability of these models in real-world healthcare systems.

The comparison of various YOLO variants further highlighted high precision of 99.17% and an F1 score of 96.95% for pharmaceutical pill recognition systems in real-world conditions (Ramlee et al., 2024). Such methods mainly focus on object localization and recognition, without integrating OCR or metadata retrieval. The systems, which use YOLOv5s for detection, ResNet for classification, and OCR for imprint recognition, obtain 97.8% recognition in pill identification tasks (Kavitha and Madhumathy, 2025). Despite these frameworks being close to a real-world healthcare system, which faces architectural challenges, requires substantial computational resources, and imposes a practical gap on lightweight edge-driven deployment.

### 2.5 Gaps and limitations of existing approaches

Despite significant improvements in AI-assisted pill recognition technologies, several implementation gaps remain insufficiently addressed in the current literature. Existing studies are limited to certain tasks, like detection (Nguyen et al., 2023; Qomariah et al., 2026; Pan et al., 2025; Sun et al., 2024; Kwon et al., 2021) or classification (Nguyen et al., 2026; Jha et al., 2025) or optical character recognition (OCR) (Wu and Chang, 2025; Jo et al., 2026; Ghazaryan et al., 2025) without developing a unified multimodal recognition system. Detection-based systems frequently lack downstream verification mechanisms (Lee et al., 2025; Wang et al., 2024; Ramlee et al., 2024). In contrast, recognition techniques based on classification typically focus on visual features such as pill shape and color, which can reduce the reliability of visually similar pills.

In addition, various multimodal frameworks incorporate computationally expensive models, such as graph neural networks (Nguyen et al., 2023) and attention-based models, thereby limiting their scalability. Thus, there is a strong need to develop lightweight architectures for pill recognition systems and practical real-world deployments.

Most existing studies were evaluated on smaller datasets with minimal variability in lighting, viewing angles, and device specifications. Evaluation of cross-device reliability and real-world implementation, including smartphone-based image capture and various input data, remains underexplored in the importance of real-time medication safety systems.

Few existing studies have attempted to incorporate pill recognition systems into medical informatics settings and AI-enabled decision support platforms (Sachin and Karthikeyan, 2026; Kavitha and Madhumathy, 2025). Consequently, there remains a need for unified, lightweight frameworks that integrate multi-pill detection, multi-attribute classification, OCR-driven imprint recognition, and metadata retrieval within a scalable, end-to-end architecture suitable for real-world healthcare implementation.

## 3 Proposed hybrid pill recognition framework

### 3.1 Overall framework architecture

An end-to-end artificial intelligence-based approach in the proposed framework automates pill identification through multi-pill detection, multi-attribute classification, imprint recognition, and metadata retrieval. The unified framework combines deep learning models for object detection. classification, Optical Character Recognition (OCR), and structured metadata retrieval within a single architecture, allowing a supportive approach to medication safety and AI-driven pharmacovigilance (Nagar et al., 2025).

The overall process begins with acquiring images from a set of customized benchmark datasets. To assess framework robustness in diverse acquisition conditions, additional cross-device analyses were performed using direct image uploads and smartphone-captured images. These analyses were designed to evaluate the effects of variations in image quality, illumination, noise, and device characteristics on Framework performance. The YOLOv26n detection model processes the acquired input image to localize multiple pills in a single frame. The detector creates bounding boxes for each pill instance, enabling multi-pill recognition across different imaging conditions and visual environments.

Following localization, each detected pill region is automatically cropped and then sent to the classification module, which is based on MobileNetV4-ConvSmall. The multi-attribute classification pipeline predicts pill class, shape, and color simultaneously, using structured labels derived from the metadata repository using a CSV file. The lightweight architecture allows computationally efficient inference without compromising high recognition performance for visually identical pills. (Kim et al., 2025).

In parallel, EasyOCR is used to extract alphanumeric imprint information from detected pill regions. The extracted imprint information serves as an additional verification during pill identification and complements the visual predictions generated by the classification module. To improve recognition reliability, OCR processing is applied only to cropped pill regions generated by the detection stage, thereby reducing background interference during imprint extraction.

After detection, classification, and OCR inference, the framework subsequently retrieves structured medication information from a CSV-based metadata repository, including medication attributes and contextual information. The retrieved metadata is integrated with prediction outputs to support medication verification and improve interpretability.

Specifically, the metadata retrieval module employs a rule-based exact-match lookup strategy in which the predicted pill class generated by the classification module is used as the primary retrieval key. The corresponding metadata record is then retrieved from the CSV-based repository to provide medication attributes, including pill name, dosage strength, shape, color, and usage information. OCR-derived imprint information is subsequently used as an auxiliary verification cue to support consistency between visual predictions and retrieved metadata. The metadata repository is implemented as a lightweight local CSV-based backend, enabling efficient offline retrieval without requiring external database services or cloud connectivity.

**Figure 1:**
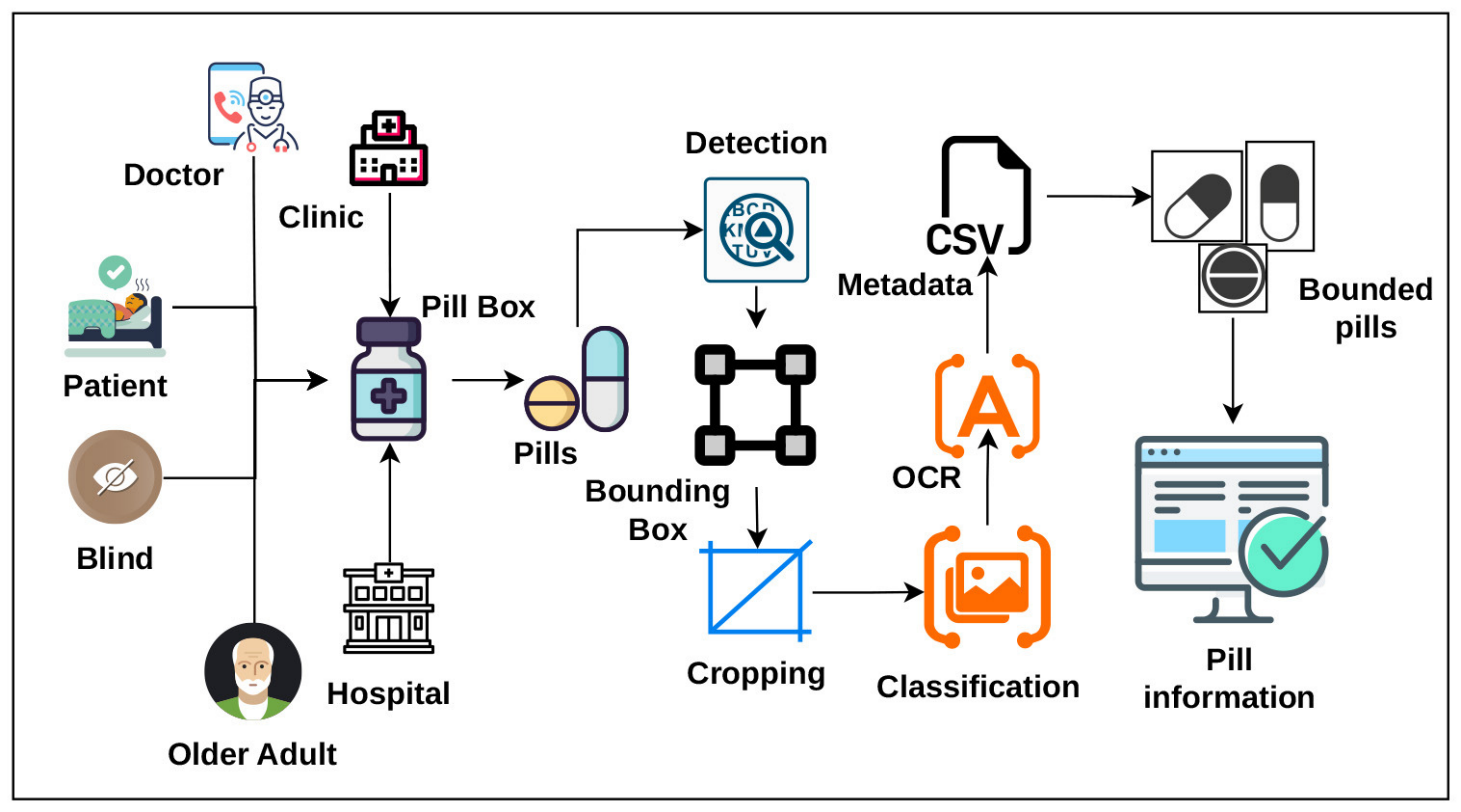
The end-to-end flow diagram demonstrating detection, classification, OCR recognition, and metadata retrieval prediction workflow.

The overall framework architecture, illustrated in Figure 1, was designed using lightweight deep learning components to support computationally efficient inference and scalable deployment. In addition, the modular design allows flexible integration within AI-based healthcare, medication safety, and pharmacovigilance support systems.

### 3.2 Multi-pill detection module

**Figure 2:**
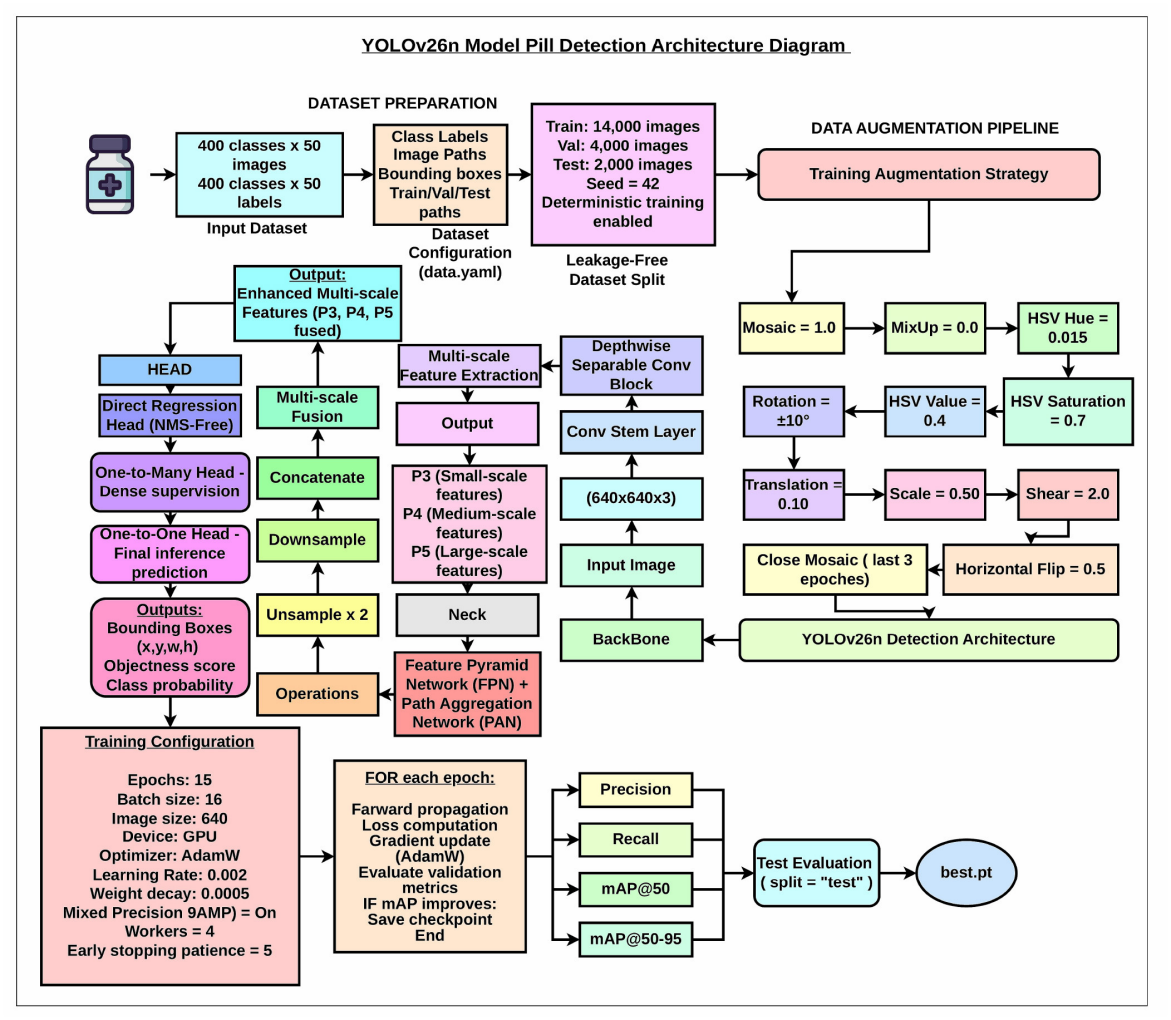
Pill detection training pipeline using YOLOv26n architecture.

The pill detection module was developed to accurately localize individual pills within an image before classification and imprint recognition. Detection represents the initial phase of the proposed framework and enables multi-pill processing in heterogeneous imaging conditions, as illustrated in Figure 2.

A lightweight YOLOv26n-based object detection architecture was used for its balanced detection accuracy and computational efficiency. YOLOv26n simultaneously localizes objects and estimates confidence, making it reliable for real-time healthcare computer vision applications and scalable deployment scenarios. The architecture uses conventional feature extraction and multi-scale prediction attributes to enable reliable detection on different sizes and multiple conditions.

Given an input image *I* ∈ ℝ^*H*×*W*×3^, the detector predicts a set of bounding boxes, as defined in Equation (1)


$$\begin{array}{l} B={\left\{\left(x_{i},y_{i},w_{i},h_{i},c_{i}\right)\right\}}_{i=1}^{N} \end{array}$$ (1)

where (*x*_*i*_, *y*_*i*_) denote the center coordinates, (*w*_*i*_, *h*_*i*_) represent the width and height of the predicted bounding box (Kang and Park, 2026), and *c*_*i*_ denotes the detection confidence score.

The detection confidence is defined in Equation (2) as follows:


$$\begin{array}{l} c_{i}=P\left(\text{object}\right)\times IoU_{pred,gt} \end{array}$$ (2)

where *P*(object) represents the objectness probability and *IoU*_*pred, gt*_ denotes the intersection-over-union between the predicted and ground-truth bounding boxes.

The detection model was initialized with weights fine-tuned on a custom pill dataset, which contains 40,000 manually labeled pill instances in 20,000 images. The model was trained using a standardized preprocessing and augmentation pipeline designed to improve robustness and generalization in heterogeneous imaging conditions. To prevent data leakage, the dataset was divided into training, validation, and testing subsets before model training.

Training was performed using the AdamW optimizer with automatic mixed precision (AMP) enabled to improve optimization stability, computational efficiency, and numerical reliability during training. The overall optimization objective consisted of bounding box regression, objectness prediction, and classification losses is formulated in Equation (3):


$$\begin{array}{l} L_{total}=\lambda_{box}L_{box}+\lambda_{obj}L_{obj}+\lambda_{cls}L_{cls} \end{array}$$ (3)

During inference, prediction bounding boxes were filtered using a confidence threshold to remove low-confidence detections and retain reliable pill localizations. The final detected pill regions were subsequently cropped and forwarded to the downstream classification and imprint recognition module for detailed pill identification.

### 3.3 Multi-attribute classification module

**Figure 3:**
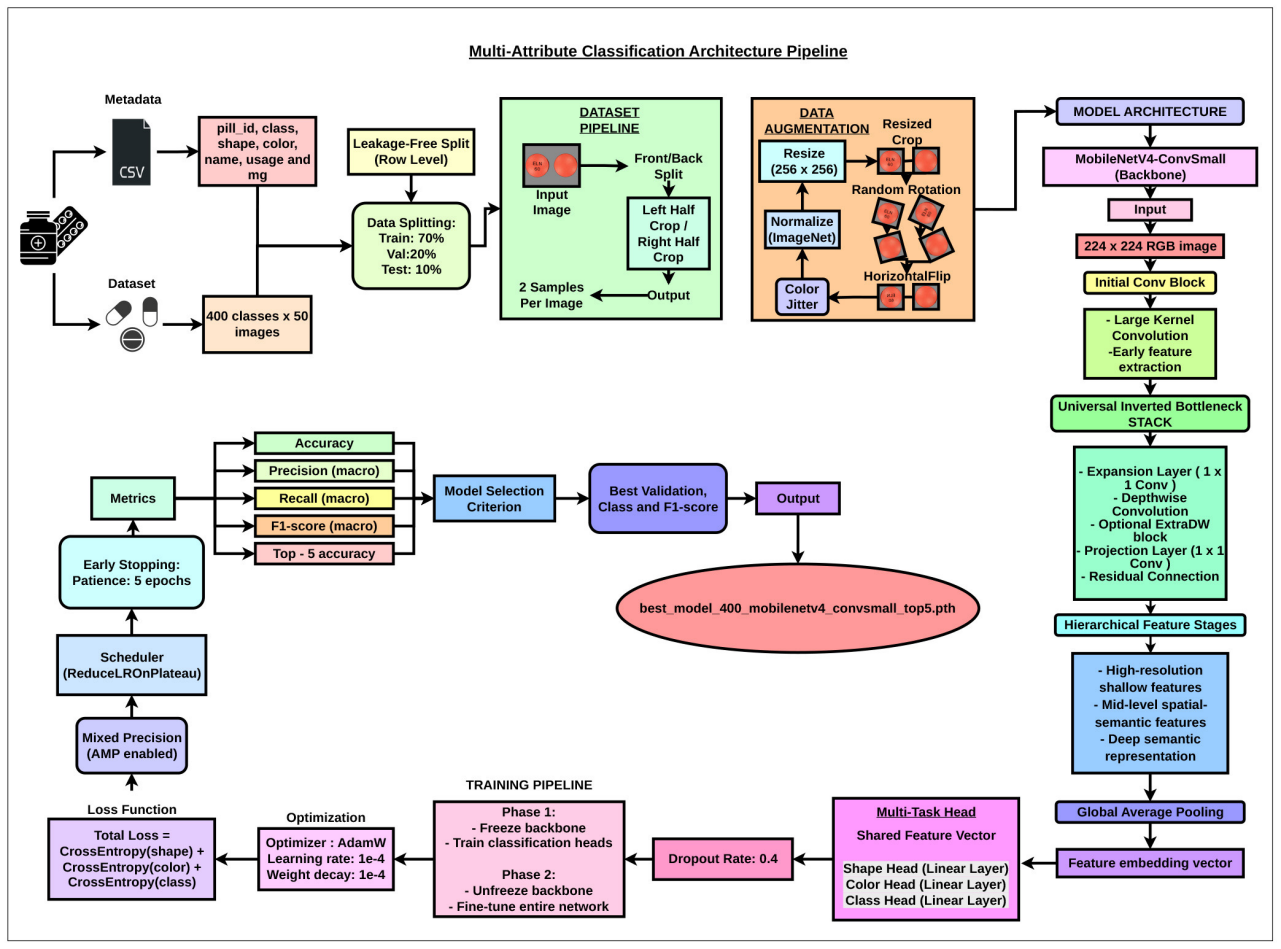
Multi-attribute classification pipeline using Lightweight MobileNetV4-ConvSmall Architecture.

The multi-attribute classification module was developed to perform a simultaneous classification of pill class, shape, and color attributes from the detected pill regions generated by the detection phase. To support computationally efficient inference under lightweight deployment constraints, MobileNetV4-ConvSmall was adapted as the backbone architecture, as illustrated in Figure 3. The model was selected due to its favorable balance between feature representation capability and computational efficiency, making it suitable for scalable healthcare-based computer vision applications.

Given an input pill image *x* ∈ ℝ^*H*×*W*×3^, the backbone network extracts a high-level feature representation, as expressed in Equation (4):


$$\begin{array}{l} f=F_{\theta }\left(x\right) \end{array}$$ (4)

where *F*_θ_ denotes the MobileNetV4-ConvSmall feature extractor and *f* represents the learned embedded feature. Dropout regularization was applied during training to improve generalization and reduce overfitting.

The proposed framework performs simultaneous multi-attribute classification of pill shape, class, and color using independent classification heads attached to the shared backbone representation, as presented in Equation (5):


$$\begin{array}{l} {ŷ}_{shape},{ŷ}_{color},{ŷ}_{class}=G\left(f\right) \end{array}$$ (5)

where *G* denotes the set of classification heads used for multi-attribute classification from the shared feature representation. This shared feature learning technique enables efficient extraction of discriminative visual characteristics while minimizing additional computational overhead.

To improve representation diversity and increase the number of training samples, a front-back image splitting strategy was applied during preprocessing. Since each benchmark image contains two pills, individual pill regions were separated into independent training samples before model training. This technique effectively increased dataset diversity while preserving leakage-free dataset separation between training, validation, and testing subsets.

The dataset was partitioned into training, validation, and test subsets prior to model development to prevent information leakage. The class, shape, and color labels were generated using the CSV-based metadata repository. Training a two-stage optimization strategy that begins with an initial backbone-freezing stage, followed by fine-tuning the entire network to improve adaptation to specific visual features of the pill.

The overall optimization objective was defined as the integrated multi-task classification loss is defined in Equation (6):


$$\begin{array}{l} L_{total}=L_{shape}+L_{color}+L_{class} \end{array}$$ (6)

where each component corresponds to the categorical cross-entropy loss for the associated classification task. The model was trained with the AdamW optimizer, and automatic mixed precision (AMP) was enabled to improve computational efficiency and numerical stability, both of which are needed for training. To prevent overfitting and rapid convergence, adaptive learning rate scheduling and early stopping were also used.

Input images were processed using a standardized preprocessing pipeline to ensure consistency during training and inference.

During inference, the final classification output was generated using the softmax decision rule, while a Top-5 accuracy evaluation was additionally performed to assess the robustness of the classification for the large-scale pill recognition task. The best-performing model checkpoint was selected based on validation macro F1-score performance and subsequently integrated into the final pill identification framework.

### 3.4 Integrated prediction pipeline

**Figure 4:**
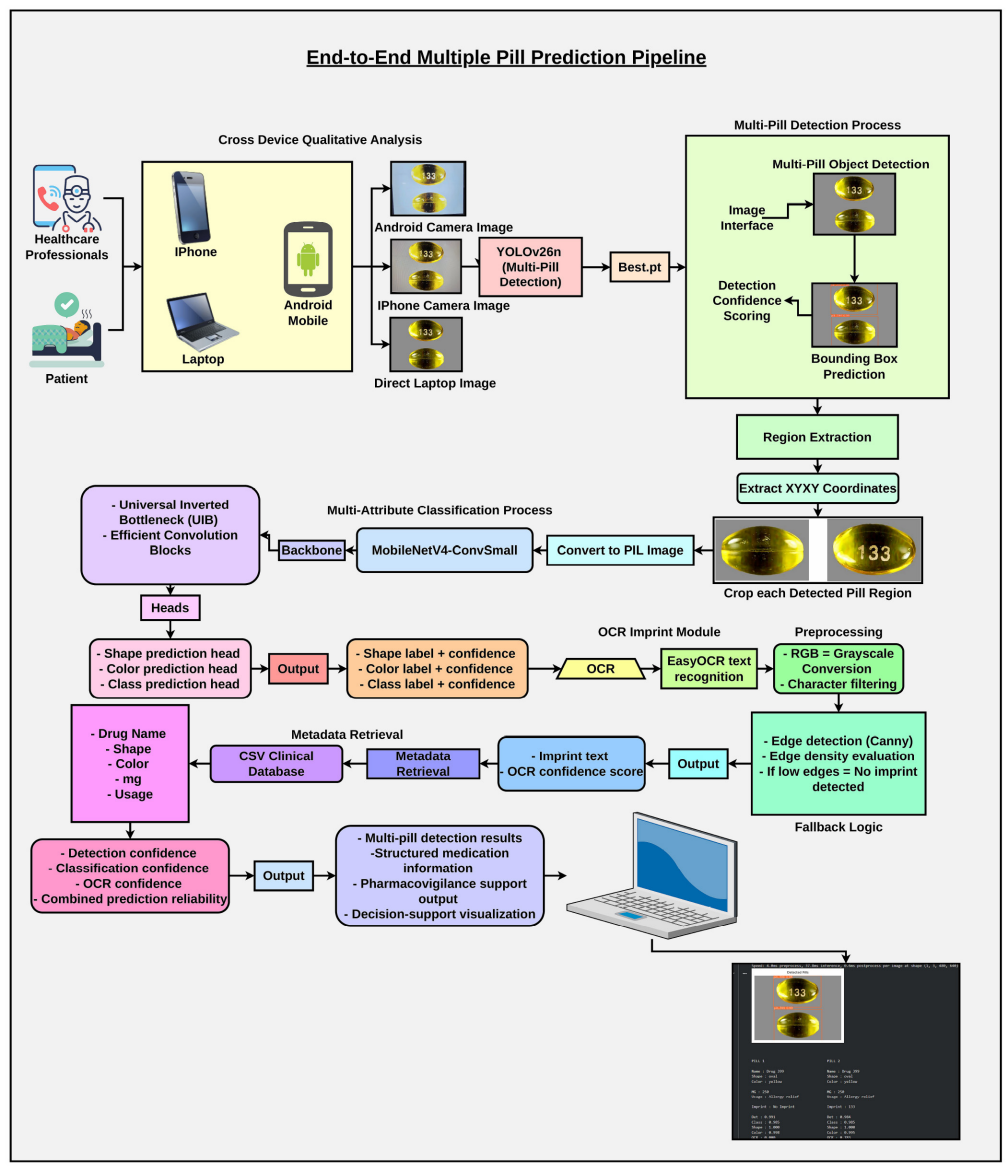
End-to-end framework demonstrating Pill detection, classification, OCR recognition, and Metadata Retrieval Workflow.

The integrated prediction framework combines multi-pill detection, multi-attribute classification, OCR-based imprint recognition, and metadata retrieval within a unified end-to-end framework for automated pill identification, as illustrated in Figure 4. The pipeline was designed to support medication safety and pharmacovigilance workflow supported by artificial intelligence in heterogeneous imaging conditions.

The overall framework can be represented by Equation (7):


$$\begin{array}{l} Y=F\left(I\right)=\left\{f_{det},f_{cls},f_{ocr},f_{meta}\right\}\left(I\right) \end{array}$$ (7)

where *F* denotes the integrated prediction pipeline consisting of detection, classification, OCR-driven imprint recognition, and metadata retrieval module operating sequentially in the input image *I* to generate the final pill identification output *Y*.

Given an input image *I* ∈ ℝ^*H*×*W*×3^, the framework initially performs pill localization using the YOLOv26n detection model, as described in Equation (8):


$$\begin{array}{l} B=f_{det}\left(I;\theta_{d}\right) \end{array}$$ (8)

where *B* = *b*_1_, *b*_2_, ..., *b*_*n*_ represents the set of pill bounding boxes θ_*d*_ detected and denotes the trained detection parameters.

Each detected pill region is subsequently cropped and forwarded to the multi-attribute classification module, as shown in Equation (9):


$$\begin{array}{l} C_{i}=Crop\left(I,b_{i}\right) \end{array}$$ (9)

where *C*_*i*_ denotes the cropped region corresponding to the *i*-th detected pill instance.

The cropped pill regions are normalized and processed using the MobileNetV4-ConvSmall classification network to perform a simultaneous classification of pill class, shape, and color attributes, as described in Equation (10):


$$\begin{array}{l} \left({ŷ}_{shape},{ŷ}_{color},{ŷ}_{class}\right)=f_{cls}\left(C_{i};\theta_{c}\right) \end{array}$$ (10)

where ŷ_*shape*_, ŷ_*color*_, and ŷ_*class*_ represent the classified shape, color, and pill category outputs, respectively.

In parallel, the alphanumeric imprint information is extracted from the cropped pill areas using OCR-driven imprint recognition with EasyOCR. Symbols that are not valid or alphanumeric are removed from the detected text before the final imprint extraction, as described in Equation (11):


$$\begin{array}{l} T=Filter\left(OCR\left(C_{i}\right)\right) \end{array}$$ (11)

where *T* represents the text of the imprinted image after the OCR module. OCR-based imprint recognition complements visual appearance by providing additional imprint information that improves differentiation between visually identical pills.

Following classification and imprint recognition, the framework retrieves medication-related information from the CSV-based metadata repository. The retrieved metadata is integrated with the prediction outputs of the detection, classification, and OCR modules to support interpretable pill identification and contextual verification.

The final product displays the detected pill instance along with metadata and confidence information, including detected pill regions, classified pill attributes, imprint-recognition results, and contextual medication information. The integrated workflow enables scalable, computationally efficient pill identification suitable for AI-based healthcare support systems and a lightweight deployment conditions.

## 4 Dataset preparation and pre-processing

### 4.1 Dataset collection, annotation, and metadata construction

Customized large-scale pill datasets were developed using the publicly available benchmark medical dataset from the U.S. Department of Health and Human Services' Pillbox (Sachin and Karthikeyan, 2026; National Library of Medicine, 2021). The Pillbox database has a total of 8,691 unique pill types, along with their pharmaceutical information. In our analysis, 400 distinct pill variations were selected to create a customized benchmark dataset of pill types commonly encountered in real-world pill identification.

Multiple Pill variants were divided into multiple pill attributes for multi-attribute classification. In this process, each pill class was expanded to 50 images using appropriate preprocessing and data augmentation approaches to increase dataset variability and improve generalization. The customized dataset contained 400 pill classes, with each class containing a total of 50 images. Thus, the total number of images used to train the AI-driven pill identification model was 20,000. Each image contains two pills; therefore, the total number of pill instances in the dataset was 40,000. The primary need for the multiple visually similar pill variations was to provide realistic challenges for AI models' pill recognition.

As shown in Table 1, the pill dataset exhibits substantial variation in the texture, shape, color, and imprint of the pill. These differences were intentionally preserved to enhance the proposed pipeline's generalization across multiple imaging conditions.

**Table 1:** Summary of the customized pill dataset and annotation statistics.

Dataset property	Value	Description
The original pill types	8,691	The pillbox benchmark dataset
The selected pill variants/classes	400	Customized benchmark subset
An augmented images per class	50	Generated images for each pill class
Total images	20,000	A complete customized dataset size
Total pill instances	~40,000	Two pills instances in each image
Shape categories	8	Distinct pill shape attributes
Color categories	39	Distinct pill color attributes
Imprint availability	Yes	An alphanumeric pill imprint information
Annotation type	Bounding Boxes	The manual object annotations
Metadata format	CSV	Structured metadata repository

Manual labeling was performed using the LabelImg annotation tool (Tan et al., 2021) to generate bounding-box labels for training the detection model. The 40,000 pill instances have been manually labeled to ensure precise pill localization and accurate ground-truth supervision for the YOLOv26n detection model.

In multi-attribute classification, each pill instance was annotated using three attribute groups, such as pill class, shape, and color. The dataset contains 400 pill classes corresponding to unique pill categories, 8 shape variants, and 39 color variants derived from the structured metadata repository. These attribute labels were used to support the simultaneous prediction of pill class, shape, and color within the proposed multi-attribute classification framework. The shape variants include round, oval, square, triangle, trapezoid, rhomboid, pentagon, and teardrop. Color annotations were derived from 39 unique color variants represented within the metadata repository, including both single-color and multi-color pill appearances.

The structured metadata representing pill variations has been stored in a CSV-based repository that includes pill identifiers, medication names, class labels, shape and color attributes, dosage information, and medication usage descriptions. This repository supports both multi-attribute classification and medication information retrieval during framework inference. Each metadata record is indexed using a unique pill identifier associated with the corresponding classification label, enabling efficient exact match retrieval of medication attributes during prediction.

### 4.2 Leakage-free dataset splitting strategy

To avoid potential bias in model evaluation and reduce information leakage (Amanatidis et al., 2025) during development, the initial dataset was split into training, validation, and test sets prior to model training. The dataset uses a stratified process, assigning 70% of the samples to training, 20% to validation, and 10% to testing, maintaining class balance in all splits.

Since each benchmark image contains two pills, front-back splitting (Zeng et al., 2017) was applied to extract additional pill samples for training. To avoid contamination between datasets, pill samples extracted from the same parent image were assigned to the same subset of the overall dataset during the split procedure. Deterministic random seeds were applied to maintain reproducibility and experimental consistency.

### 4.3 Image pre-processing and data augmentation

To improve input consistency and model generalization, standardized preprocessing and data augmentation procedures were applied during model development. For object detection, all images were resized to 640 × 640 pixels and bounding-box annotations were converted to the YOLO annotation format. For multi-attribute classification, pill regions obtained through the front-back splitting strategy were resized according to the input requirements of the MobileNet architectures and normalized using ImageNet (Usuyama et al., 2020) statistics during training and inference.

Several controlled augmentation techniques (Dang et al., 2024) were applied during training, including random rotation, translation, scaling, horizontal flipping, color jittering, HSV-based color perturbation, and mosaic augmentation for object detection. HSV augmentation was used to simulate illumination variability and improve robustness under heterogeneous imaging conditions. Mixup augmentation was intentionally excluded because combining multiple pill instances could alter clinically relevant visual characteristics and reduce the interpretability of pill appearance. The selected augmentation strategies were designed to preserve medically relevant visual attributes while improving robustness under real-world imaging conditions.

In general, the preprocessing and augmentation framework was designed to preserve important pill attributes, including shape, color, and imprint information, while improving model robustness across diverse acquisition devices and imaging environments.

## 5 Experimental setup

### 5.1 Experimental environment

All experiments were conducted using a Python-based deep learning pipeline for multi-pill detection, multi-attribute classification, OCR recognition, and integrated inference. The proposed framework was implemented using PyTorch (Wang et al., 2026b) and associated deep learning libraries to enable scalable experimentation and reproducible evaluation. The object detection component was developed using the Ultralytics (Sapkota and Karkee, 2025) YOLO framework, while multi-attribute classification models were implemented using the timm library for lightweight neural network deployment. OCR-driven imprint recognition experiments were performed using EasyOCR, TesseractOCR (Paglinawan et al., 2023), and TrOCR (Zaman et al., 2024) models for comparative analysis.

The experimental pipeline was designed with lightweight deployment considerations to support potential real-world healthcare and edge-device applications that require computationally efficient inference.

### 5.2 Hardware and software configuration

The proposed framework was developed and evaluated using a hybrid local and cloud-based experimental condition. Local preprocessing, annotation management, metadata handling, and inference-based experiments were conducted using an HP Laptop equipped with an Intel Core i5 processor and 16 GB RAM.

Computationally intensive training and large-scale experiments were performed using the Google Colab cloud platform with NVIDIA Tesla T4 GPU acceleration (Dymora et al., 2025). GPU-driven training was used for detection, classification, and OCR-based inference to improve computational efficiency during model optimization and evaluation (Sachin and Karthikeyan, 2026). In addition, optimization was implemented to improve memory utilization and accelerate training performance while maintaining numerical stability.

### 5.3 Training configuration

#### 5.3.1 Detection model training

Pill detection experiments were performed using multiple YOLO architectures, including YOLOv11n (Chen et al., 2025), YOLOv12n (Malepati et al., 2026), and YOLOv26n, to comparatively evaluate lightweight detection performance under identical experimental settings. All detection models were trained using an image resolution of 640 × 640, a batch size of 16, and 15 training epochs.

Training optimization was used with AdamW (Nsumba et al., 2026) optimizer with an initial learning rate of 0.002 and a weight decay of 5 × 10^−4^. Deterministic training with a fixed random seed was also applied to enhance reproducibility, while early stopping with a patience of 5 epochs was used to reduce overfitting.

#### 5.3.2 Multi-attribute classification training

Multi-attribute classification experiments were performed using a lightweight MobileNet architecture, including MobileNetV2-Small (Kannan et al., 2025), MobileNetV3-Small (Pinto et al., 2025), and MobileNetV4-ConvSmall to support computationally efficient implementation.

Training was conducted using input images resized to 220 × 224, a batch size of 32, and 15 training epochs. Optimization used the AdamW optimizer with a learning rate of 1 × 10^−4^ and a weight decay of 1 × 10^−4^. In addition, early stopping (Chiu, 2024) with a patience value of five epochs was added to improve generalization performance.

### 5.4 Evaluation metrics and validation strategies

To comprehensively evaluate the proposed framework, separate evaluation metrics were defined for pill detection, multi-attribute classification, OCR-driven imprint recognition, and integrated end-to-end prediction.

The detection module was evaluated using precision, recall, mean Average Precision at the IoU threshold 0.50 (mAP@50), mean Average Precision across multiple IoU thresholds (mAP@50-95), and Intersection over Union (IoU) (Wang et al., 2025). Precision and recall were computed, as defined in Equations (12) and (13):


$$\begin{array}{l} Precision=\frac{TP}{TP+FP} \end{array}$$ (12)

$$\begin{array}{l} Recall=\frac{TP}{TP+FN} \end{array}$$ (13)

where *TP*, *FP*, and *FN* denote true positive, false positive, and false negative, respectively.

Mean Average Precision at IoU threshold 0.50, as described in Equation (14):


$$\begin{array}{l} mAP@50=\frac{1}{N}\overset{N}{\underset{i=1}{\sum }}AP_{i}^{0.50} \end{array}$$ (14)

where $AP_{i}^{0.50}$ denotes the average precision of class *i* calculated as an IoU threshold of 0.50, and *N* represents the total number of classes.

Mean Average Precision across multiple IoU thresholds, as described in Equation (15):


$$\begin{array}{l} mAP@50\text{-}95=\frac{1}{10N}\overset{0.95}{\underset{t=0.50}{\sum }}\overset{N}{\underset{i=1}{\sum }}AP_{i}^{t} \end{array}$$ (15)

where $AP_{i}^{t}$ represents the average precision for the class *i* evaluated in IoU thresholds ranging from 0.50 to 0.95 with increments of 0.05.

The IoU metric used for detection evaluation was defined in Equation (16):


$$\begin{array}{l} IoU=\frac{Area\left(B_{pred}\cap B_{gt}\right)}{Area\left(B_{pred}\cup B_{gt}\right)} \end{array}$$ (16)

where *B*_*pred*_ and *B*_*gt*_ represent the predicted and ground-truth bounding boxes.

The multi-attribute classification phase was evaluated using accuracy, macro precision, macro recall, macro F1-score, and Top-5 accuracy. The Top-5 accuracy was included due to the large number of pill classes and the presence of visually identical pills.

Detection confidence (Wenkel et al., 2021) was calculated using Equation (17):


$$\begin{array}{l} Conf_{det}=P\left(object\right)\times IoU_{pred,gt} \end{array}$$ (17)

where *P*(*object*) denotes the objectness probability predicted by the detection model and *IoU*_*pred, gt*_ represents the overlap between the predicted and ground-truth bounding boxes.

The confidence score (Shen et al., 2025) for pill variants classification was calculated using the maximum softmax probability, as shown in Equation (18):


$$\begin{array}{l} Conf_{class}=max\left(Softmax\left(z_{class}\right)\right) \end{array}$$ (18)

where *z*_*class*_ denotes the classification logits corresponding to the prediction of the pill variants.

The shape classification confidence was defined using Equation (19):


$$\begin{array}{l} Conf_{shape}=max\left(Softmax\left(z_{shape}\right)\right) \end{array}$$ (19)

where *z*_*shape*_ represents the logits generated for pill shape classification.

Similarly, the color classification confidence was calculated using Equation (20):


$$\begin{array}{l} Conf_{color}=max\left(Softmax\left(z_{color}\right)\right) \end{array}$$ (20)

where *z*_*color*_ represents the logits generated for pill color classification.

OCR-driven imprint recognition performance was evaluated using imprint recognition confidence and valid imprint detection robustness under heterogeneous imaging conditions. OCR confidence (Vignesh et al., 2025) was calculated using Equation (21):


$$\begin{array}{l} Conf_{ocr}=\frac{1}{N}\overset{N}{\underset{i=1}{\sum }}p_{i} \end{array}$$ (21)

where *p*_*i*_ denotes the recognition probability of the *i*-th detected character and *N* represents the total number of recognized characters.

To assess framework robustness under different image acquisition conditions, cross-device qualitative evaluation experiments were additionally conducted using multiple image acquisition platforms. The evaluation was initially performed using direct benchmark dataset image uploads under controlled experimental conditions. Additional experiments were conducted using smartphone-driven image acquisition, where benchmark pill images displayed on a laptop screen were captured using Android and iPhone devices equipped with high-resolution camera systems.

Cross-device qualitative evaluation experiments were conducted to examine framework performance under heterogeneous acquisition conditions, including variations in camera characteristics, image quality, illumination conditions, and acquisition perspectives.

### 5.5 Framework configuration evaluation

To evaluate the integrated pipeline of lightweight detection and classification architectures on overall pill recognition performance, a comparative framework evaluation was performed. The two high performing detection models and the two high performing classification models identified during the preliminary experiments were selected to construct four framework configurations. Each configuration integrated a detection model with a classification model while maintaining identical OCR, metadata retrieval, preprocessing, and evaluation conditions.

The evaluation was performed using the same test dataset generated during dataset partitioning. The complete dataset consisted of 20,000 pill images representing 400 pill categories and was divided into training, validation, and test subsets using a 70%,20% and 10% split strategy. Consequently, all framework configurations were evaluated on the same test set comprising 1,980 images to ensure a fair and consistent comparison.

For each test image, pill localization was first performed using the selected detection model, followed by pill classification using the corresponding classification backbone. Performance was evaluated using Top-1 Accuracy, Top-5 Accuracy, Precision, Recall, and F1-score. By maintaining all remaining framework components unchanged, this evaluation protocol enabled a systematic comparison of different detector and classifier combinations and their impact on overall pill recognition performance.

## 6 Results and analysis

### 6.1 Detection performance analysis

The performance of the proposed multi-pill detection framework was evaluated using multiple lightweight YOLO architectures, including YOLOv11n, YOLOv12n, and YOLOv26n. Among the evaluated models, YOLOv26n was found to be the best in multi-pill detection settings and showed strong localization accuracy and reliability. The model achieved a precision of 0.963, a recall of 0.982, an mAP@50 of 0.989, and an mAP@50-95 of 0.981. The quantitative detection results are illustrated in Table 2.

**Table 2:** Performance comparison of lightweight YOLO architectures for pill detection.

Model	Layers	Parameters (M)	GFLOPs	Precision	Recall	mAP@50	mAP@50–95
YOLOv11n	182	2.74	7.3	0.912	0.968	0.981	0.969
YOLOv12n	272	2.72	7.3	0.934	0.979	0.986	0.975
YOLOv26n	260	2.81	7.4	**0.963**	**0.982**	**0.989**	**0.981**

Bold values indicate the best-performing results for each evaluation metric among the compared methods.

The results have shown the efficiency of the proposed detection pipeline to detect multiple pills in different visual scenarios. The high recall further indicates that the model has shown a strong performance at detecting multiple instances of pills in a single image, which is required for a real-time pill identification system.

**Figure 5:**
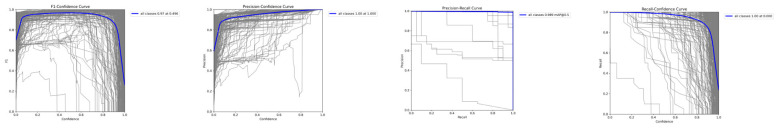
The confidence curves of YOLOv26n detection model.

The precision-confidence curve, recall-confidence curve, F1-confidence curve, and precision-recall curve (Jepisah et al., 2025) corresponding to the selected YOLOv26n detection model are shown in Figure 5. The results highlight stable convergence behavior and strong generalization performance throughout training.

**Figure 6:**
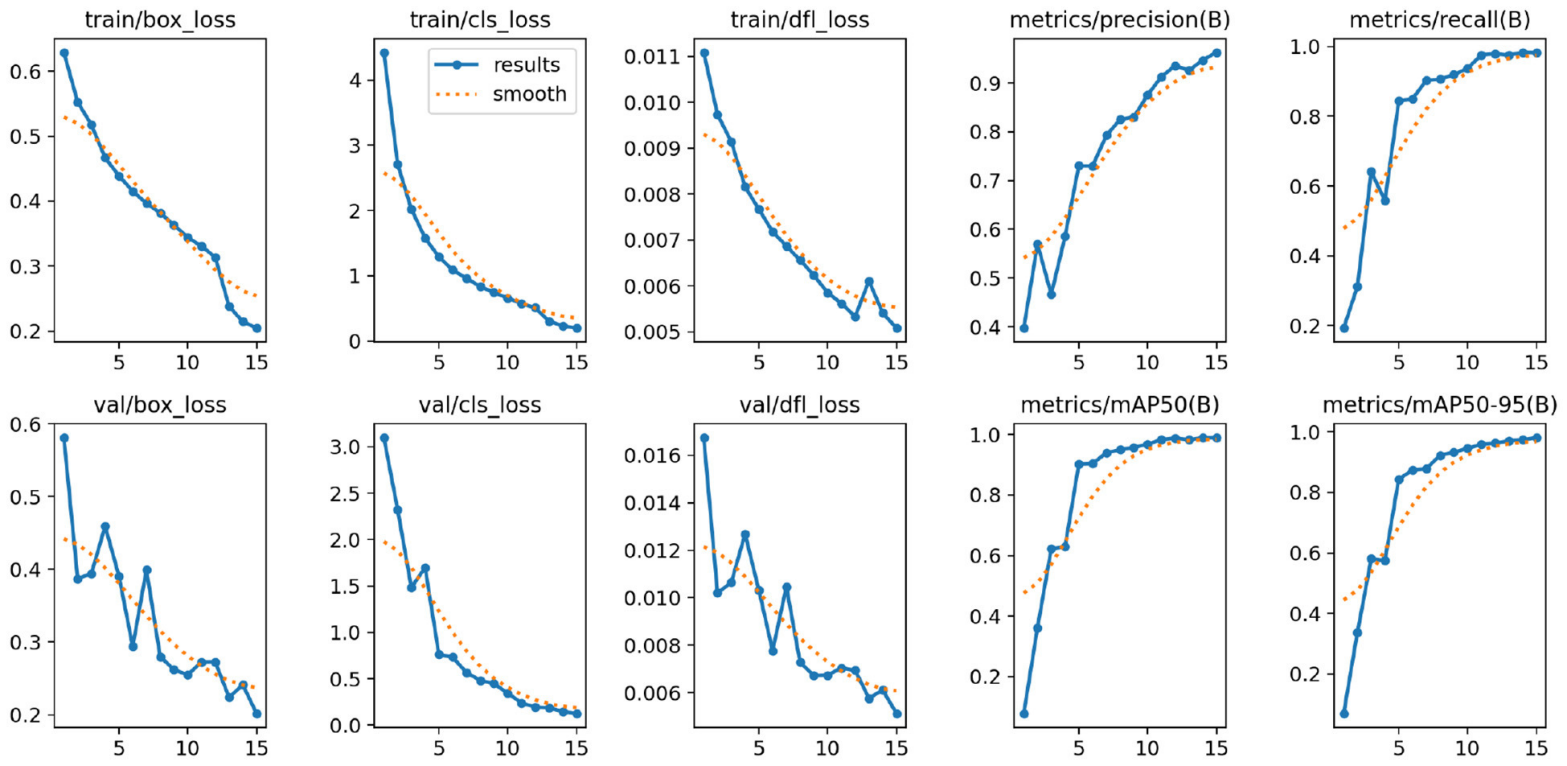
Training and validation convergence curves of YOLOv26n detection model.

Training and validation convergence curves, including box loss, classification loss, distribution focal loss (DFL) (Li et al., 2022), precision, recall, mAP@50, and mAP@50-95 in training epochs, are presented in Figure 6.

### 6.2 Multi-attribute classification performance

The proposed multi-attribute classification framework was evaluated using lightweight MobileNet architectures, including MobileNetV2-Small, MobileNetV3-Small, and MobileNetV4-ConvSmall. The comparative classification performance is summarized in Table 3.

**Table 3:** Performance comparison of lightweight architectures for multi-attribute pill classification.

Model	Task	Accuracy	Precision	Recall	F1-score	Loss
MobileNetV2-Small	Shape	0.9977	0.9996	0.9996	0.9996	0.0463
	Color	1.0000	1.0000	1.0000	1.0000	0.0463
	Class	0.9864	0.9898	0.9863	0.9854	0.0463
MobileNetV3-Small	Shape	1.0000	1.0000	1.0000	1.0000	0.0194
	Color	1.0000	1.0000	1.0000	1.0000	0.0194
	Class	0.9922	0.9938	0.9923	0.9918	0.0194
MobileNetV4-ConvSmall	Shape	1.0000	1.0000	1.0000	1.0000	0.0114
	Color	1.0000	1.0000	1.0000	1.0000	0.0114
	Class	**0.9960**	**0.9964**	**0.9956**	**0.9954**	**0.0114**

Bold values indicate the best-performing results for each evaluation metric among the compared methods.

Among the evaluated models, MobileNetV4-ConvSmall achieved the best overall performance in all classification tasks. The model achieved a higher classification accuracy for pill shape and color, and for pill class prediction, with 99.60% accuracy, 99.64% precision, 99.56% a recall, and 99.54% of F1-score in the 400-class pill classification task.

These results highlight the effectiveness of the proposed multi-head learning framework for simultaneously predicting multiple pill attributes while maintaining computational efficiency suitable for lightweight deployment environments. The representation of the shared feature further improved discriminative capability in visually similar pill classes.

### 6.3 Confusion matrix analysis

The confusion matrix (Salmon et al., 2015) was evaluated to assess class-level prediction performance and identify potential misclassification patterns in both the detection and classification modules.

**Figure 7:**
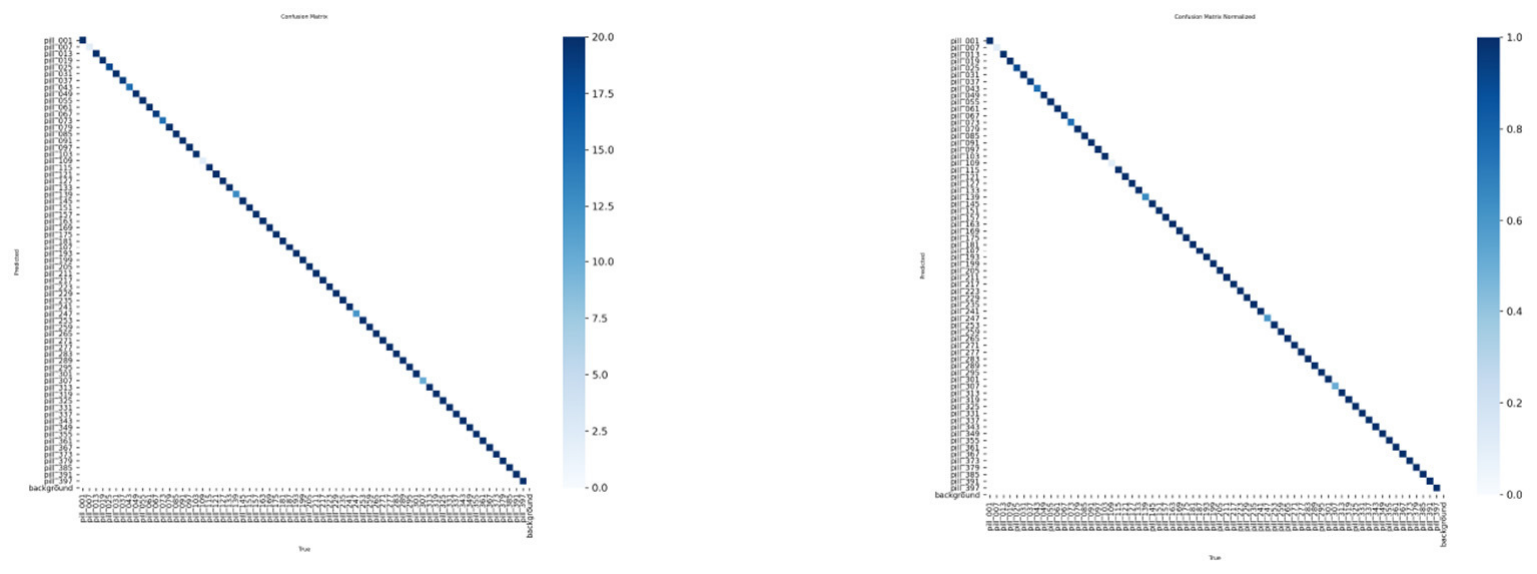
The confusion matrix of YOLOv26n detection model.

**Figure 8:**
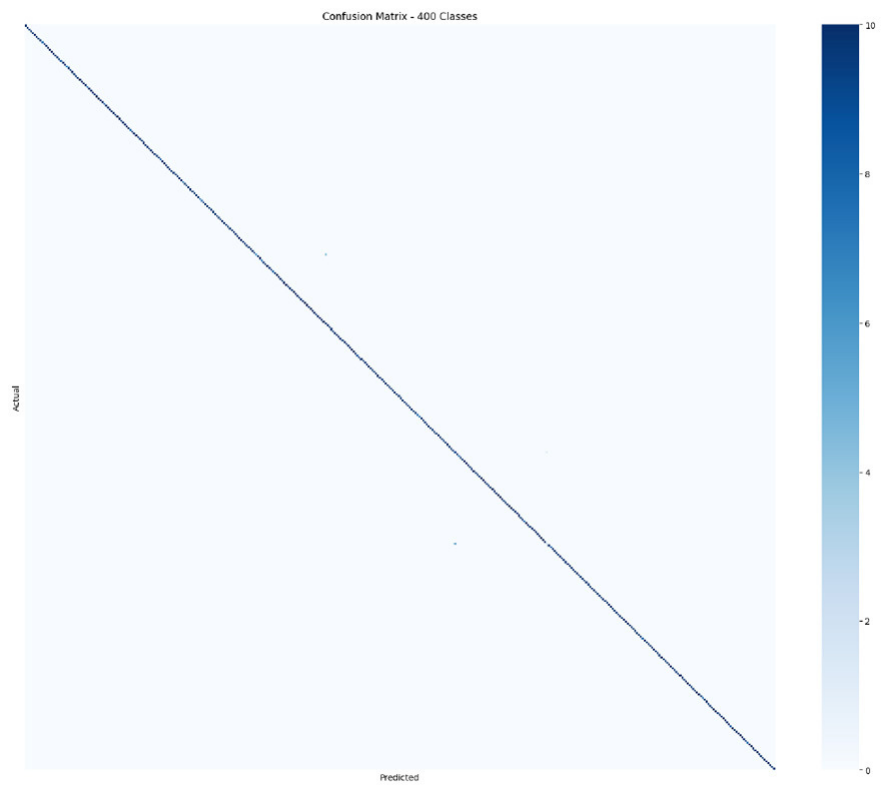
The confusion metrix of MobileNetV4-ConvSmall classification model.

The confusion matrix for the YOLOv26n object detection model is shown in Figure 7, whereas the confusion matrix for the multi-attribute classifier MobileNetV4-ConvSmall model is shown in Figure 8.

The confusion matrix results show minimal misclassification across multiple pill categories. This shows strong discrimination based on learned features, even among visually identical pills. Misclassification occurred between pills that contain highly similar visual features, such as comparable shape and color, reflecting the complexity of large-scale pill recognition, which involves thousands of pill classes.

In general, the results show that the utilization of multi-attribute learning strategies improves classification performance when combined as shape, color, and pill classes.

### 6.4 OCR imprint recognition performance

The OCR-driven imprint recognition module was evaluated using multiple OCR pipelines, including EasyOCR, TesseractOCR, and TrOCR. Comparative experiments were conducted to evaluate recognition reliability under varying imprint visibility and heterogeneous conditions.

**Figure 9:**
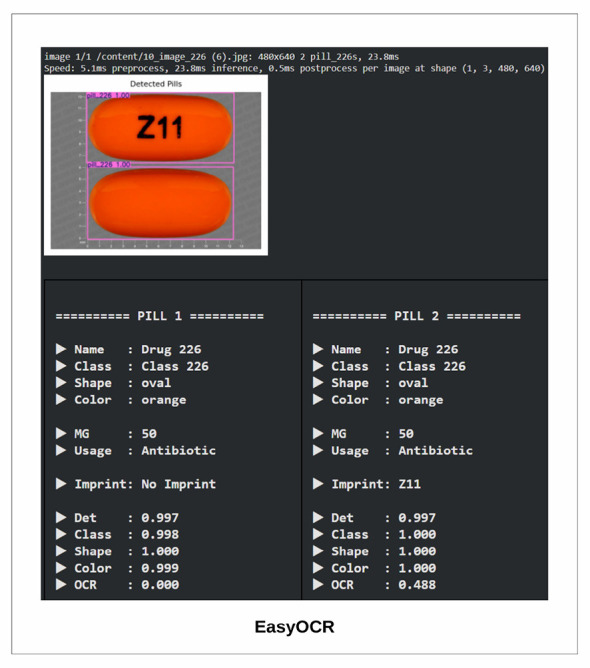
EasyOCR qualitative evaluation.

**Figure 10:**
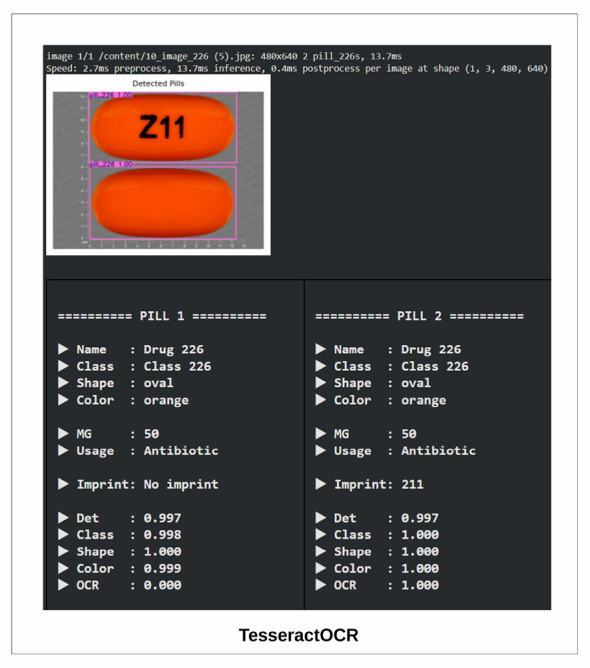
TesseractOCR qualitative evaluation.

**Figure 11:**
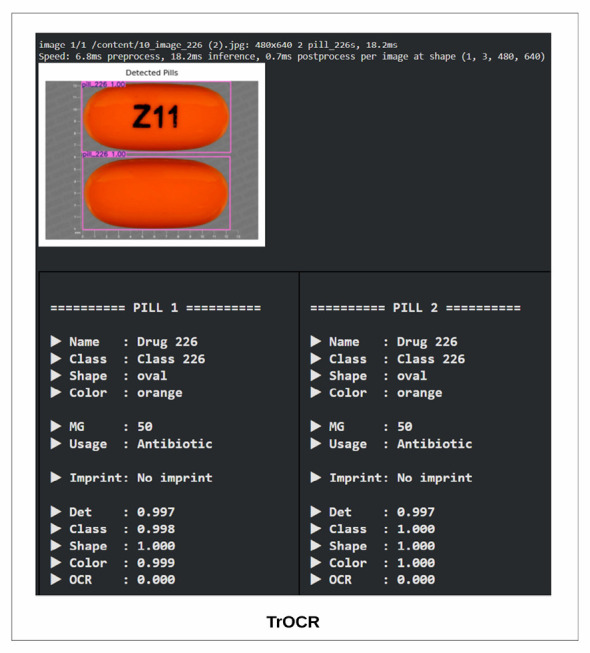
TrOCR qualitative evaluation.

Among these, the EasyOCR qualitative OCR comparison provided more reliable recognition for short alphanumeric pill imprints. For example, the imprint “Z11” was correctly recognized by EasyOCR, whereas TesseractOCR incorrectly predicted the imprint as “211,” and TrOCR failed to produce a valid recognition output under the same imaging conditions. These observations demonstrate the effectiveness of EasyOCR for practical pill imprint recognition involving small, visually challenging alphanumeric markings. The comparative OCR prediction examples are illustrated in Figures 9–11.

In addition, OCR confidence scores were analyzed to assess recognition consistency in varying image conditions. The results further support the suitability of EasyOCR for practical pill imprint recognition.

### 6.5 End-to-end multi-pill prediction performance

The overall performance of the framework was analyzed using an end-to-end approach that included detecting multiple pills, classifying multiple attributes, OCR for imprint recognition, and retrieving metadata.

**Figure 12:**
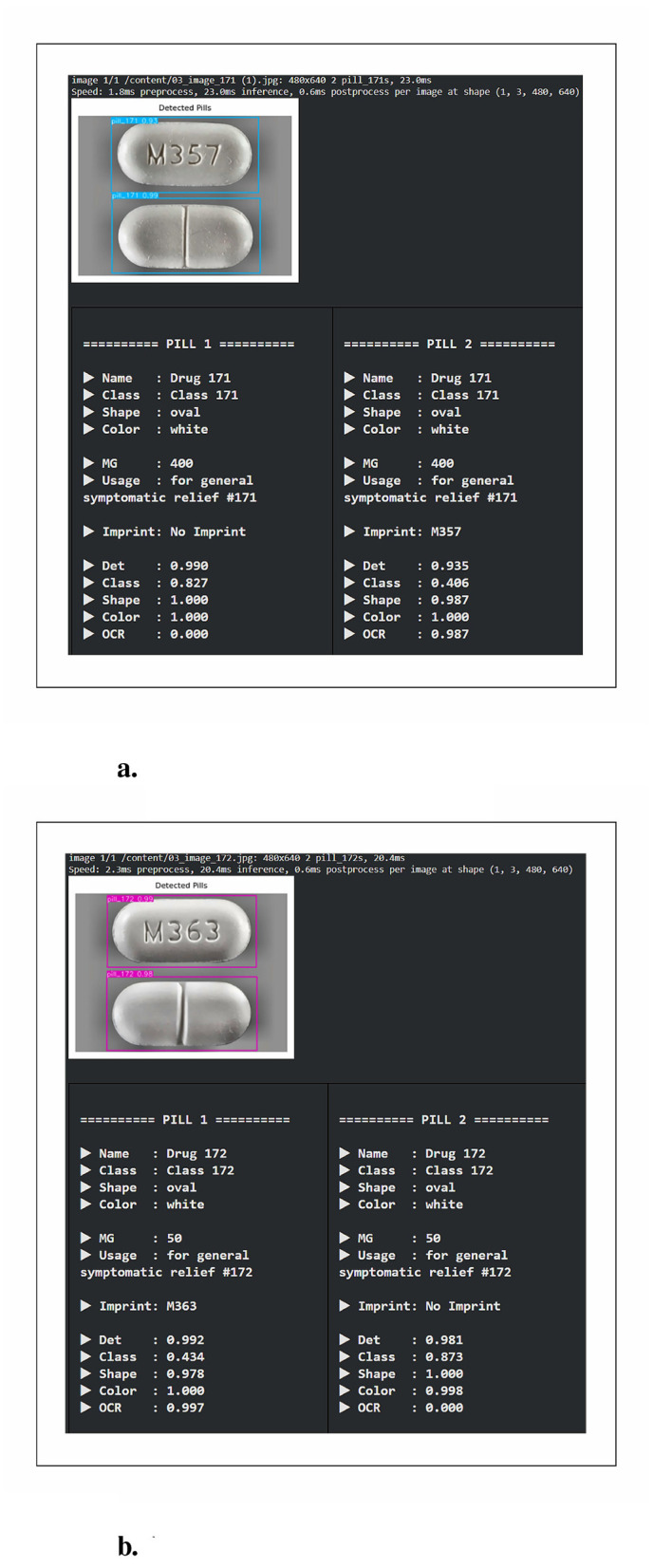
Reliable recognition of visually similar pills through the proposed framework. **(a)** Visually Similar Pill A. **(b)** Visually Similar Pill B.

The end-to-end prediction framework demonstrated strong recognition of visually identical and visually similar pills by combining pill localization, shape, color, class predictions, and imprint information, as illustrated in Figures 12a, b.

The combination of multiple prediction modules provides complementary information for pill identification in challenging multi-pill scenarios. This capability is important in medication safety settings, where visually similar pills can cause medication errors.

### 6.6 Cross-device qualitative evaluation

To evaluate framework reliability across heterogeneous acquisition environments, cross-device experiments were conducted using benchmark pill images acquired through smartphone-based imaging approaches (Agu et al., 2013) (Hunt et al., 2021). A representative pill image from the benchmark dataset was captured through Android smartphones, iPhones, and direct laptop uploads to qualitatively assess framework behavior in different image acquisition conditions.

Images captured using Android devices demonstrated stable framework performance under varying illumination conditions and imaging perspectives, achieving an inference time of 54.8 ms and an FPS of 14.95. Similarly, iPhone-acquired images produced reliable detection, multi-attribute classification, and OCR-based imprint recognition results, with an inference time of 113.2 ms and an FPS of 8.58.

Under controlled conditions, direct laptop image uploads consistently achieved high detection, classification, and OCR confidence, with an inference time of 109.0 ms and an FPS of 8.97.

Although the predicted pill identities remained consistent across the evaluated acquisition platforms, variations in confidence scores were observed. These variations are likely associated with differences in camera characteristics, image quality, illumination conditions, and acquisition perspectives across the evaluated devices, which can influence the confidence levels produced by the detection, classification, and OCR modules.

In general, the experiments indicate that the proposed framework maintains stable performance across different acquisition platforms despite variations in imaging conditions.

**Figure 13:**
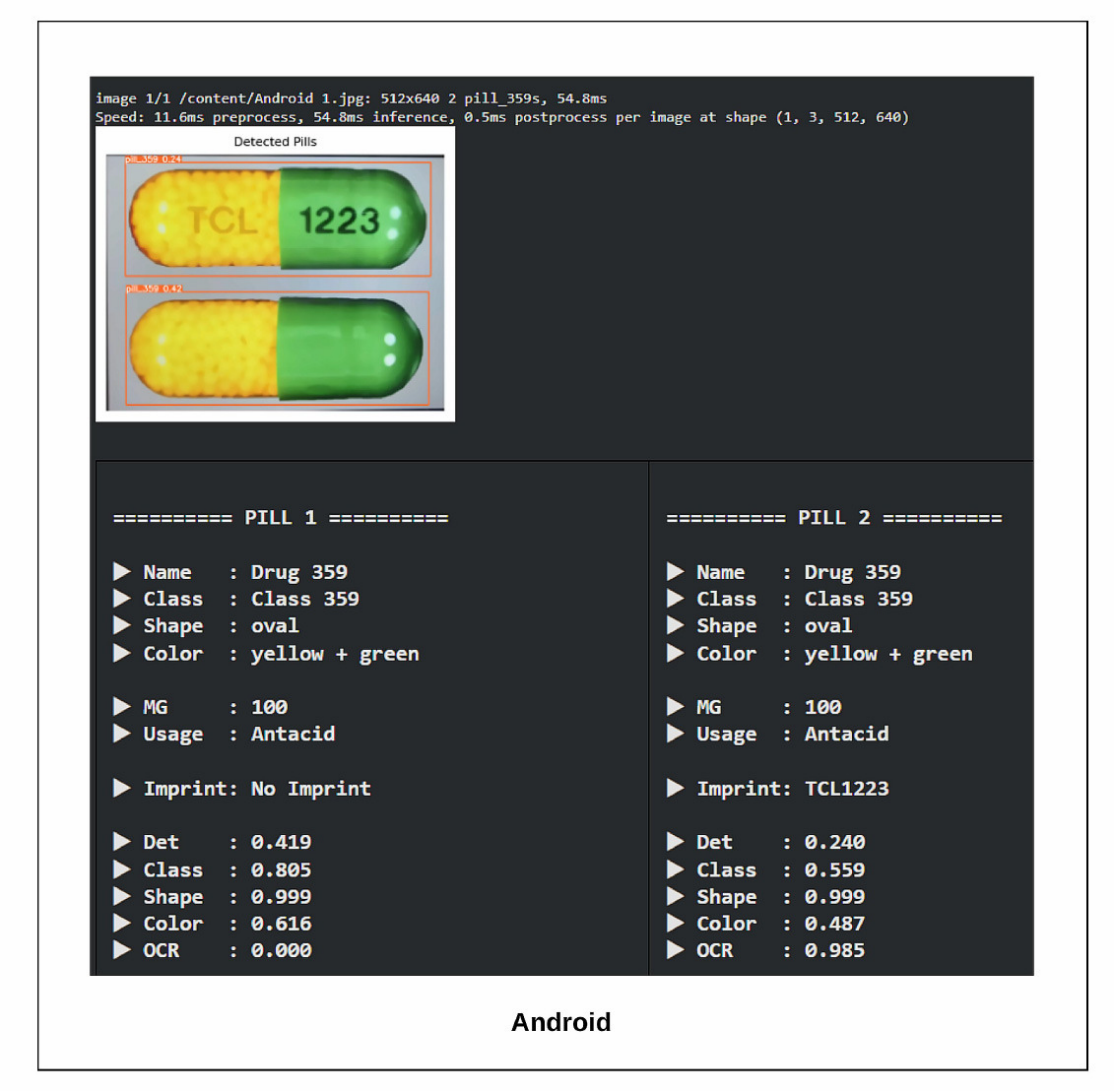
Android smartphone-based evaluation.

**Figure 14:**
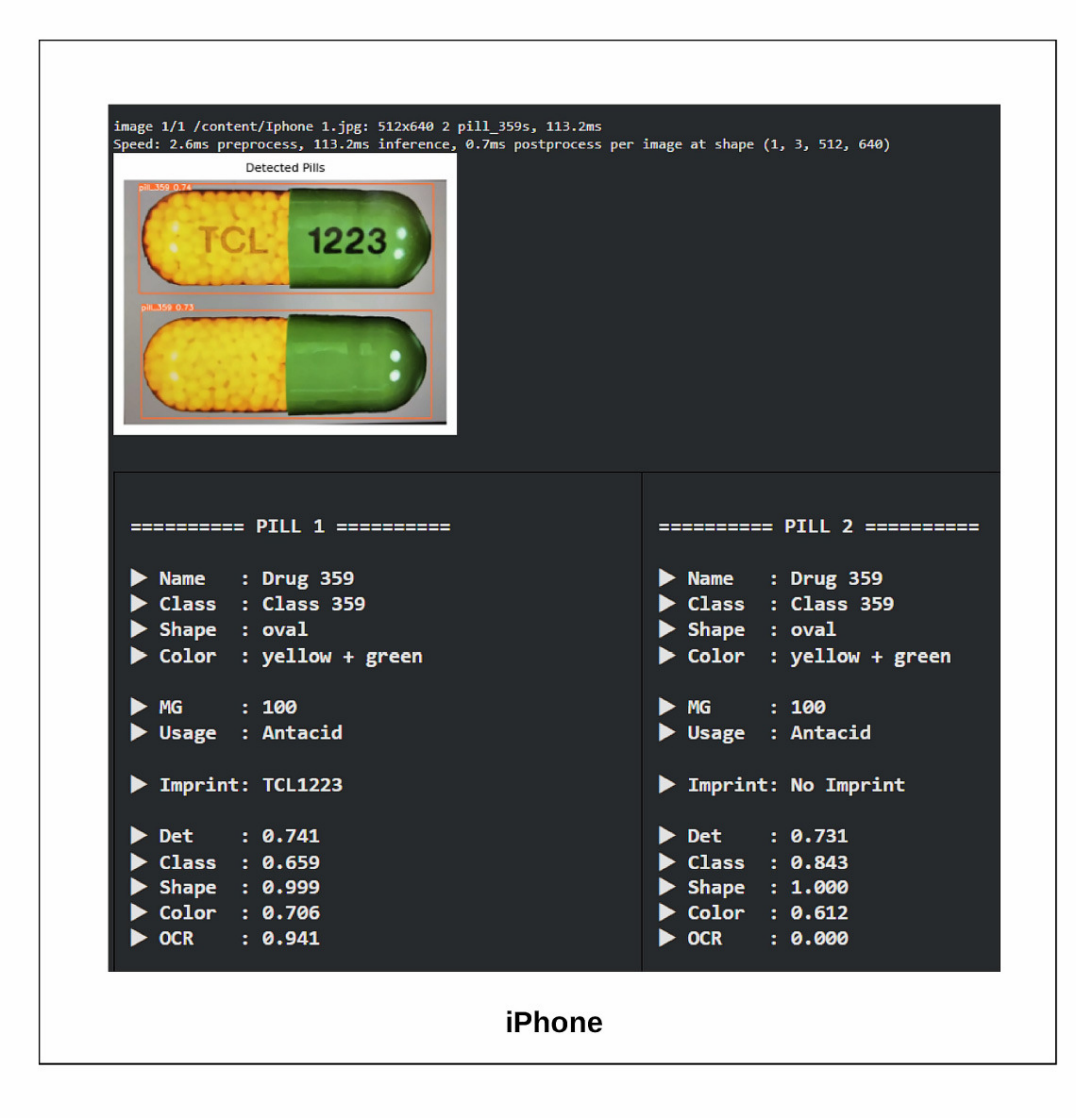
iPhone-based evaluation.

**Figure 15:**
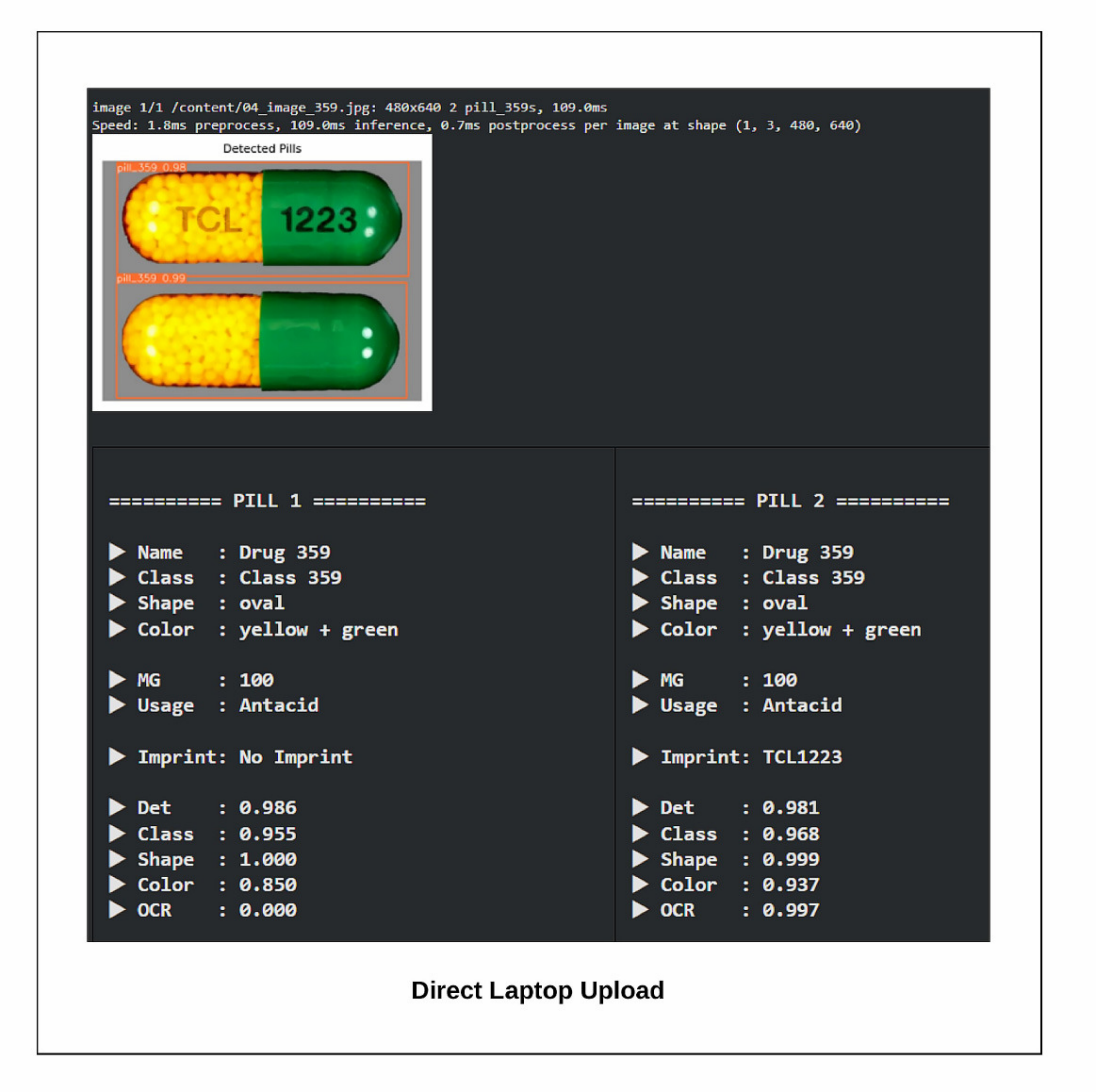
Direct laptop upload-based evaluation.

Representative prediction outputs obtained from Android devices, iPhones, and direct laptop uploads are presented in Figures 13–15.

### 6.7 Functional comparison with existing pill recognition systems

The proposed framework integrates multi-pill detection, multi-attribute classification, OCR-based imprint recognition, and metadata retrieval within a single lightweight architecture. To position the proposed framework within the current literature, a functional comparison was conducted with existing pill recognition systems. The comparison was performed based on four major framework capabilities, such as pill detection, pill classification, OCR-based imprint recognition, and multi-pill recognition.

As summarized in Table 4, many existing studies primarily focus on individual tasks, such as pill detection, classification, or OCR-based imprint analysis, while only a limited number of approaches attempt to integrate multiple functionalities within a single framework. In contrast, the proposed framework combines these complementary capabilities into an end-to-end pill recognition pipeline, enabling comprehensive pill identification through simultaneous localization, multi-attribute classification, and imprint recognition. This integrated design enhances practical applicability in real-world healthcare environments and addresses several limitations associated with task-specific pill recognition systems.

**Table 4:** Functional Comparison with existing pill recognition systems.

Study	Source	Detection	Classification	OCR	Multi-pill
Multi-pill recognition	(Lee et al., 2025)	✓	✓		✓
Real-time pill image recognition	(Nguyen et al., 2026)		✓		
Pill recognition	(Ramlee et al., 2024)	✓	✓		
Hybrid deep learning framework	(Sachin and Karthikeyan, 2026)	✓	✓		
Stacked pillbox detection	(Pan et al., 2025)	✓			
Pill detection for medicine inspection	(Kwon et al., 2021)	✓			
**Proposed framework**		✓	✓	✓	✓

Bold values indicate the best-performing results for each evaluation metric among the compared methods.

### 6.8 Comparative performance analysis of framework configurations

A comparative evaluation was performed to investigate the influence of different lightweight detector and classifier combinations on overall pill recognition performance. The quantitative results achieved from the four framework configurations are summarized in Table 5.

**Table 5:** Performance comparison of framework configurations.

Framework configuration	Top-1	Top-5	Precision	Recall	F1-score
YOLOv12n + MobileNetV3-Small	87.96	98.74	90.20	87.80	86.84
YOLOv12n + MobileNetV4-ConvSmall	89.12	99.39	90.82	89.09	87.63
YOLOv26n + MobileNetV3-Small	88.15	98.63	90.17	88.15	87.09
YOLOv26n + MobileNetV4-ConvSmall	**89.83**	**99.49**	**91.06**	**89.78**	**88.38**

Bold values indicate the best-performing results for each evaluation metric among the compared methods.

Among the evaluated Frameworks, YOLOv26n with MobileNetV4-ConvSmall achieved the highest overall performance, achieving a Top-1 accuracy of 89.83%, Top-5 accuracy of 99.49%, precision of 91.06%, recall of 89.78%, and an F1-score of 88.38%. The results illustrate that both the detection and classification architectures contributed to overall recognition performance. MobileNetV4-ConvSmall consistently achieved better performance than MobileNetV3-Small when paired with both YOLO variants, while YOLOv26n achieved better overall performance than YOLOv12n across all evaluated metrics.

**Figure 16:**
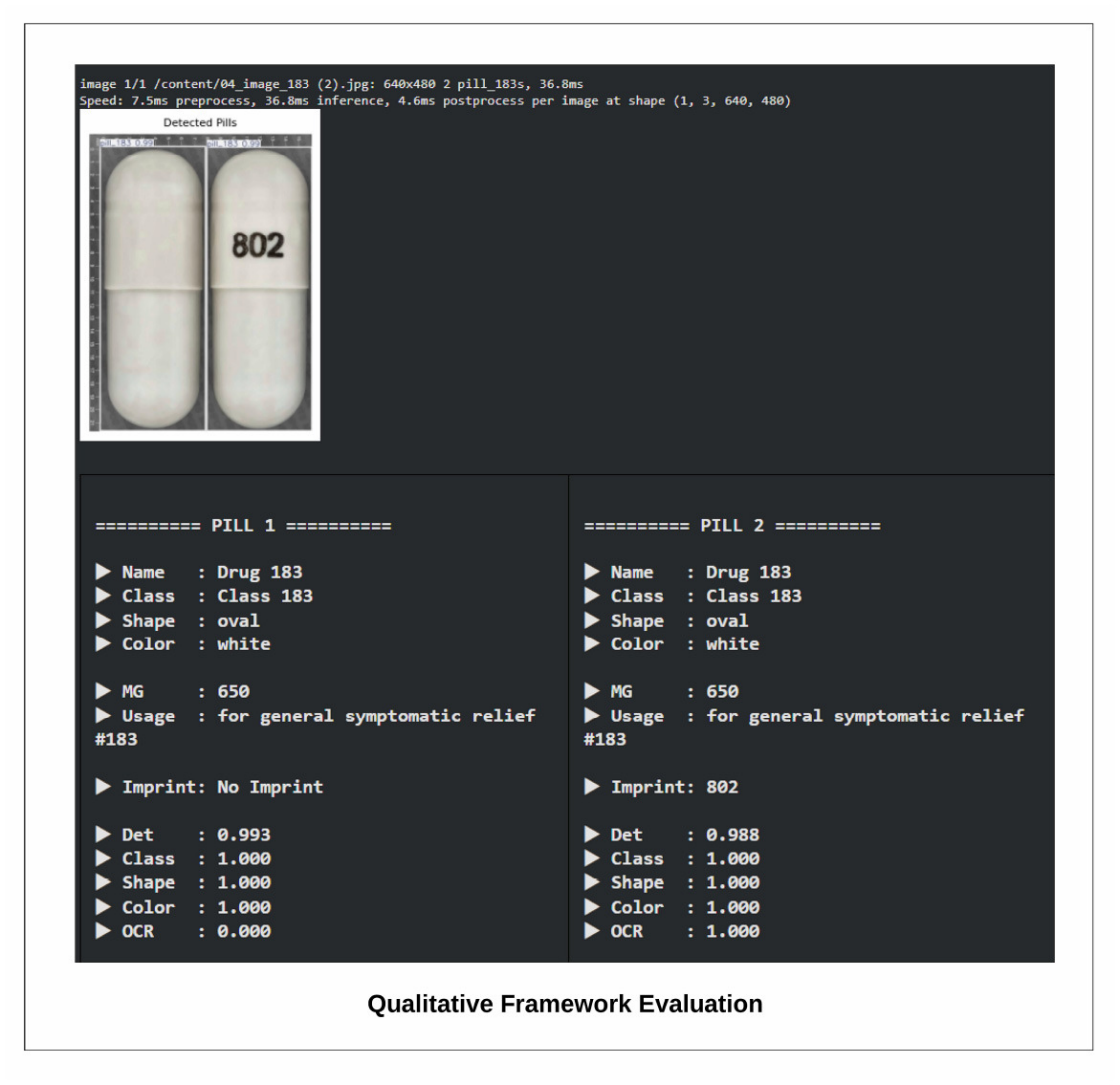
Qualitative framework evaluation. Representative prediction outputs produced by the proposed framework configuration are presented to show the framework's detection performance, multi-attribute classification capability, OCR-driven imprint recognition, and metadata retrieval functionality in identical input conditions.

A representative qualitative comparison of the four framework configurations is presented in Figure 16. Although all configurations successfully recognized the target pill category, differences in prediction confidence and prediction ranking were observed. Overall, the YOLOv26n with MobileNetV4-ConvSmall configuration demonstrated the most reliable and consistent performance and was therefore selected as the final framework for the proposed pill recognition system.

## 7 Discussion

### 7.1 Overall framework effectiveness

The experimental results highlight that the proposed lightweight framework obtained robust performance in multi-pill detection, multi-attribute classification, and OCR-based imprint recognition tasks. The combination of complementary modules within a unified pipeline improved robustness for practical multi-pill identification settings. In addition, a large-scale, manually labeled dataset contains 40,000 pills in 400 pill categories, enabling robust learning across various visual features.

The YOLOv26n detection model demonstrated effective localization capability for multiple pills in various scenes, while the multi-attribute classification pipeline using MobileNetV4-ConvSmall improved discriminative performance through combined classification of pill class, shape, and color attributes. OCR-driven imprint recognition provided additional imprint information that assisted differentiation between visually similar pills using alphanumeric imprint characteristics.

The framework-level comparative evaluation further demonstrated that the combination of YOLOv26n and MobileNetV4-ConvSmall achieved the highest overall recognition performance among the evaluated lightweight framework configurations. The results indicate that improvements in both detection and classification architectures contributed positively to overall pill recognition performance. MobileNetV4-ConvSmall consistently achieved better classification performance than MobileNetV3-Small, while YOLOv26n provided more reliable pill localization performance than YOLOv12n. These findings support the selection of YOLOv26n and MobileNetV4-ConvSmall as the final components of the proposed pill recognition framework.

### 7.2 Clinical and practical relevance

Reliable pill identification plays a crucial role in reducing medication errors, thereby supporting pharmacovigilance workflows and reducing Adverse Drug Events (ADEs) in healthcare applications. The incorrect identification of medication is a major contributor to the risk of patient safety, particularly in polypharmacy environments, where the pills are visually similar.

The proposed end-to-end pipeline contributes to medication safety by combining automated pill detection, classification, imprint recognition, and metadata retrieval as a lightweight framework.

In addition, metadata retrieval improves interpretability by providing medication names, dosages, and usage information, thereby supporting a safer medication verification process for healthcare professionals, caregivers, and patients.

### 7.3 Strengths of the proposed framework

Several strengths were observed throughout the experimental evaluation of the proposed framework. First, the framework combines multi-pill detection, multi-attribute classification, OCR-based imprint recognition, and metadata retrieval into a unified prediction framework rather than as isolated single-task predictions.

Second, by combining the attributes of pill shape, color, and class, the multi-attribute learning strategy improved the robustness of recognition for visually identical pills.

Third, cross-device qualitative evaluation demonstrated the ability of the framework to operate across heterogeneous image acquisition scenarios, including smartphone-captured images and benchmark images.

Finally, the lightweight characteristic of the proposed framework is primarily attributed to the need of computationally efficient model architectures and a low-overhead metadata retrieval mechanism, allowing efficient deployment without relying on computationally intensive backbone networks or external database services.

### 7.4 Limitations

Despite promising experimental performance, several limitations remain within the proposed framework. The system depends primarily on visual pill characteristics and OCR-derived imprint information, which may be affected by poor illumination, low-resolution image acquisition, motion blur, occlusion, or partially degraded pill characteristics.

Although OCR-driven imprint recognition improved discrimination between visually identical pills, recognition confidence can still degrade when imprint text is unclear, faded, or partially visible. Similarly, pills with highly comparable visual appearance can occasionally produce ambiguous predictions under challenging imaging conditions.

In addition, the OCR module was evaluated primarily through comparative imprint recognition analysis and OCR confidence assessment. Character-level evaluation metrics such as Character Error Rate (CER) and Word Error Rate (WER) were not investigated because the customized benchmark dataset did not contain manually annotated character-level ground-truth imprint transcriptions.

In addition, the framework was evaluated using a customized subset derived from a benchmark pharmaceutical dataset under controlled experimental conditions. Therefore, further large-scale validation using diverse real-world pill datasets and heterogeneous acquisition settings is necessary to fully assess practical generalizability and reliability.

The current cross-device evaluation was limited to a representative benchmark image and therefore does not provide a comprehensive quantitative assessment of device-specific generalization.

Furthermore, the proposed framework was developed and evaluated using a supervised learning setting involving predefined pill categories. Consequently, the current study does not investigate recognition of previously unseen pill classes, open-set pill identification, or out-of-distribution generalization. The ability to recognize novel medications that were not included during training remains an important challenge for future research.

### 7.5 Future directions

Future work can focus on extending the proposed framework to edge healthcare systems based on the Internet of Things (IoT) (Kashani et al., 2021) for the identification and validation of real-world pills. Integration with lightweight embedded devices can further support practical implementation in mobile and resource-constrained healthcare conditions.

Additional validation experiments under multiple lighting conditions, heterogeneous backgrounds, and various real-world acquisition settings can further improve reliability and generalizability. Future research can investigate large real-time pill datasets that contain categories in pill appearance and imaging conditions.

Future studies may investigate large-scale cross-device evaluations using diverse unseen pill images, multiple smartphone models, acquisition environments, and user conditions to quantitatively assess framework robustness and generalization.

Future research can also incorporate dedicated imprint recognition datasets containing character-level annotations to facilitate quantitative OCR evaluation using metrics such as Character Error Rate (CER) (Pujari et al., 2026) and Word Error Rate (WER) (Zaman et al., 2024). Such evaluation protocols can provide a more comprehensive assessment of OCR performance in challenging imprint visibility conditions.

In addition, future studies can investigate open-set (Li et al., 2025) and few-shot (Chee et al., 2024) pill recognition approaches capable of identifying previously unseen pill categories and improving framework generalization under real-world deployment settings.

Furthermore, Explainable Artificial Intelligence (XAI) (Saraswat et al., 2022) techniques can be incorporated to improve model interpretability and provide transparent decision-support information to healthcare professionals and end users. Clinical validation studies and integration with healthcare decision-support systems can support the practical adoption of AI-assisted pill recognition technologies for pharmacovigilance, medication safety, and reduction of Adverse Drug Events (ADEs).

## 8 Conclusion

This study presented a lightweight end-to-end multi-pill recognition framework integrating YOLOv26n-based pill detection, MobileNetV4-ConvSmall multi-attribute classification, OCR-based imprint recognition, and metadata retrieval within a unified prediction framework. The manually annotated dataset supported large-scale multi-pill detection and recognition experiments. The proposed framework was designed to support practical pill identification workflows while maintaining computational efficiency suitable for lightweight and edge-based deployment conditions. The experimental results highlighted robust performance in pill detection, classification, and imprint recognition tasks under heterogeneous imaging conditions. The proposed multi-attribute learning strategy improved discriminative capacity through combined classification of pill class, shape, and color attributes. In addition, OCR-based imprint recognition provided additional imprint information to assist the identification of visually similar pills. Metadata retrieval further improved interpretability by providing structured pharmaceutical information, including medication name, dosage, and usage description. Comparative framework evaluation demonstrated that the combination of YOLOv26n and MobileNetV4-ConvSmall achieved the highest overall recognition performance among the evaluated lightweight framework configurations, supporting its selection as the final framework architecture. Cross-device qualitative evaluation using smartphone-driven image acquisition and benchmark dataset uploads demonstrated the ability of the framework to operate across heterogeneous image acquisition conditions. In general, the proposed framework demonstrated strong potential to support pharmacovigilance workflows, reduce medication errors, and minimize the risk of Adverse Drug Events (ADEs) in real-world healthcare settings. The lightweight architecture and integrated prediction design further support the future deployment of AI-assisted pill identification systems in real-world healthcare settings.

## Data Availability

Publicly available datasets were analyzed in this study. This data can be found here: https://datadiscovery.nlm.nih.gov/Chemicals-and-Drugs/Pillbox-Archived-Data/crzr-uvwg/about_data.
